# A Concise Overview of Studies on Successful Real-World Applications of Bacteriophages in Aquaculture

**DOI:** 10.3390/v16121843

**Published:** 2024-11-28

**Authors:** Jin Woo Jun

**Affiliations:** Department of Aquaculture, Korea National University of Agriculture and Fisheries, Jeonju 54874, Republic of Korea; advancew@af.ac.kr; Tel.: +82-63-238-9253

**Keywords:** antibiotic resistance, bacteriophage, aquaculture, aquatic animal, therapy, prophylaxis, biocontrol

## Abstract

Increasing antibiotic resistance poses an urgent global public health threat and a serious concern worldwide. Bacteriophage (phage) therapy has been identified as a promising alternative to antibiotics for treating bacterial diseases in both humans and animals. The excessive use of antibiotics in aquaculture is a major threat to sustainable aquaculture, promoting the spread of antibiotic resistance in the aquaculture environment and the contamination of aquaculture products with antibiotic residues. Consequently, interest in alternative approaches that reduce reliance on antibiotics has grown within the aquaculture sector. As a promising alternative, extensive phage research targeted at aquaculture has demonstrated the protective efficacy of phages against diseases in aquatic animals. Although numerous studies have employed in vitro models, research supported by in vivo experiments remains scarce. Without in vivo evidence, phage therapy cannot fulfill the requirements of aquaculturists. The first part of this review outlines the bacterial diseases severely affecting the health and survival of aquatic animals. The second part provides updates on phage applications for the therapy and prophylaxis of pathogenic bacterial infection in aquatic animals, including administration routes and key accomplishments. Therefore, this review provides insights into effective real-world phage biocontrol strategies that enable sustainable aquaculture.

## 1. Introduction

Bacteriophages, abbreviated as phages, are viruses that infect and kill bacteria. They outnumber bacteria by a factor of ten and represent the most abundant organisms on Earth, estimated to number 10^31^ [1]. Phages play a critical role in maintaining microbial balance within ecosystems [2]. Phages were discovered by Frederick Twort and Félix d’Hérelle in 1915 and 1917, respectively. Frederick Twort postulated the existence of phages but could not substantiate his theory. Félix d’Hérelle, from the Pasteur Institute, recognized as the pioneer of phage therapy, successfully isolated living particles that could reproduce and undergo lysogenization. He later introduced the term “bacteriophage” [3].

**Figure 1:**
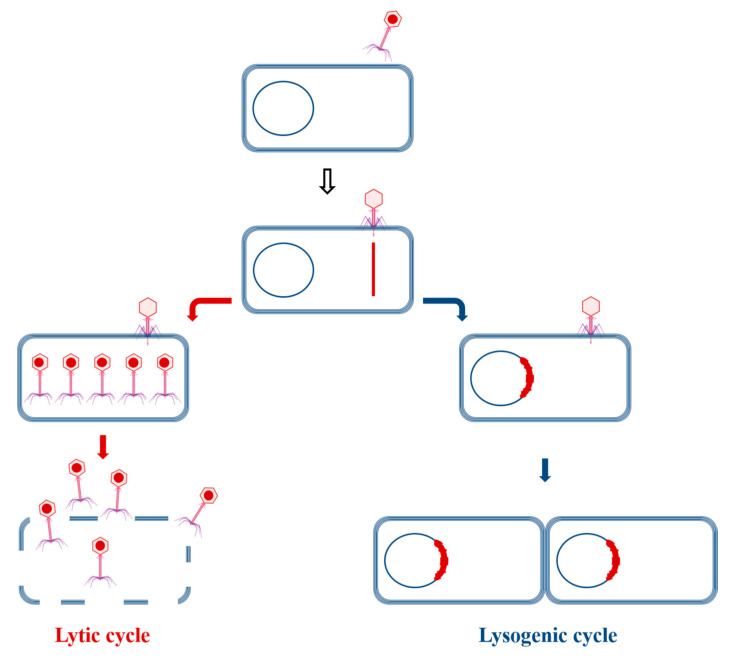
Lytic and lysogenic replication cycles of phages. Virulent phages demonstrate a lytic cycle, while temperate phages follow a lysogenic cycle.

Phages are divided into two groups based on their replication cycles: lytic and lysogenic (Figure 1). Phages that exhibit only a lytic replication cycle are termed virulent phages. Lytic phage infection initiates with adsorption to specific receptors on the bacterial host surface. Following infection, lytic phages hijack the metabolic machinery of bacteria to replicate themselves and synthesize new phage particles. Progeny phage particles are then released from the host cell through lysis, leading to the death of the host and enabling new phage particles to initiate another lytic cycle. Lytic phages are employed in treating bacterial diseases, a method known as phage therapy. Conversely, temperate phages undergoing a lysogenic cycle do not immediately destroy host cells. During a lysogenic cycle, phage DNA is injected into the cell, and some genes are expressed without producing viral progeny. The viral nucleic acid integrates into the bacterial genome, remaining in prokaryotic cells as prophages. Prophages can exist in host cells in a plasmid form. This state persists until the lytic cycle is triggered by certain stress factors such as antibiotic treatment or DNA damage [4,5].

Phage therapy was initially implemented in 1919 [6]. The application of phage therapy expanded during the 1920s to address a broad range of human infections, including cholera, dysentery, bubonic plague, conjunctivitis, and skin infections [6,7]. In 1923, Giorgi Eliava and Félix d’Hérelle established the G. Eliava Institute of Bacteriophages, Microbiology and Virology in Tbilisi, Georgia. This institute has long been involved in developing and producing phages for the treatment and prevention of bacterial infections, including intestinal ailments [8]. However, the 1930s saw the publication of several critical reviews of phage therapy trials [9]. These reviews questioned the validity of earlier results due to issues such as a lack of controls, low efficacy, possible confounders, and low purity of phage lysates [9]. Additionally, the “golden age” of antibiotics commenced in the 1940s with the introduction of penicillin and lasted until the 1970s [6]. During this period, over 40 antibiotics were discovered and introduced into clinical practice [6]. Since then, the discovery of new antibiotics has decreased, leading to the emergence of antibiotic-resistant bacteria [6]. Today, as global healthcare faces the challenge of increasing antibiotic resistance, phage therapy is once again becoming prominent [10].

Aquaculture has become the fastest-growing food production sector, yet it faces a serious challenge with the emergence and spread of diseases [11]. Generally, bacterial diseases are the most significant impediments in aquaculture, as fish in stressful environments are more susceptible to bacterial infections [12]. This has led to increased reliance on antibiotics, resulting in a rise in antibiotic-resistant bacteria in aquaculture, including pathogens affecting fish and shellfish. Phages have been employed to control bacterial infections in aquaculture, prompted by the growing demand for alternatives to antibiotics [13,14,15,16,17,18,19,20,21,22,23,24,25,26,27,28,29,30,31,32,33,34,35,36,37]. Since the first phage therapy trial for controlling fish bacterial infections in 1981, numerous studies have demonstrated phages’ effectiveness against the most destructive bacteria in aquaculture, for example, *Aeromonas* spp., *Edwardsiella* spp., *Flavobacterium* spp., *Lactococcus* spp., *Pseudomonas* spp., *Streptococcus* spp., and *Vibrio* spp. [13,14,15,16,17,18,19,20,21,22,23,24,25,26,27,28,29,30,31,32,33,34,35,36,37].

Previously, phage applications in aquatic animals were extensively reviewed, showcasing their potential as a biocontrol agent against bacterial infections [38,39,40,41,42,43,44]. This review highlights significant achievements in phage application within the field of aquatic animals, including effective treatment methods. It aims to provide insights into successful real-world phage applications in aquaculture, paving the way for sustainable practices.

## 2. Disease Control in Aquaculture

Seafood is an essential food resource worldwide. Currently, aquaculture stands as one of the leading sectors in animal production. According to the Food and Agriculture Organization (FAO) of the United Nations, global aquaculture production reached 130.9 million tons in 2022, with aquaculture and fisheries combined accounting for 59% of total production [45]. Aquaculture and its associated industries have evolved steadily, with aquaculture exceeding capture fisheries in aquatic animal production for the first time in 2022, making up 57% of the production intended for human consumption [45].

Despite developments in aquaculture, the threat of infectious diseases persists. Several unavoidable conditions in the aquaculture environment, including the high density of animals, which induces stress and hygienic issues, exacerbate this situation [42]. Various bacterial species from the genera *Aeromonas*, *Edwardsiella*, *Flavobacterium*, *Lactococcus*, *Pseudomonas*, *Streptococcus*, and *Vibrio* can cause morbidity and mortality in cultured fish and shellfish, leading to significant economic losses [43].

### 2.1. Bacterial Diseases in Aquaculture

The genus *Aeromonas* belongs to the family *Aeromonadaceae*. These primarily aquatic bacteria are widespread in water [46]. Some *Aeromonas* spp. are pathogenic to both humans and aquatic animals, with growing interest in their zoonotic potential [47]. *Aeromonas* is categorized into two groups: motile and non-motile aeromonads [47]. *A. hydrophila* is the most prevalent cause of motile *Aeromonas* septicemia, presenting as tail and fin rot with hemorrhagic septicemia [48]. Key symptoms of *A. hydrophila* infection include fin erosion, scale loss, gill hemorrhage, abdominal swelling, and ascites accumulation [48]. On the other hand, *A. salmonicida*, the only non-motile species in the genus *Aeromonas*, is the causative agent of furunculosis [49]. *A. salmonicida* affects a wide range of hosts, infecting not only salmonids but also various freshwater and marine fish [43]. Furunculosis is a major bacterial disease in global aquaculture, inflicting substantial economic losses in salmonid cultivation [49]. Acute furunculosis, which often results in fatality within days of infection, manifests as fish darkening, lethargy, appetite loss, and fin base hemorrhage. The sub-acute or chronic form is more common in older fish, presenting with reddened fins, bloody vent discharge, lethargy, eye protrusion, and the hemorrhaging of muscles and organs [48].

The genus *Edwardsiella*, previously positioned in the family *Enterobacteriaceae*, is a member of the family *Hafniaceae* [50]. Although the taxonomy of this genus is still controversial, it contains five known species: *E. anguillarum*, *E. hoshinae*, *E. ictaluri*, *E. piscicida*, and *E. tarda* [51]. Among them, *E. anguillarum*, *E. ictaluri*, and *E. piscicida* are known to cause diseases in fish [50]. It has been noted that pathogenic *Edwardsiella* species carry virulence genes such as Type III, Type IV, and Type VI secretion systems, which play a crucial role in pathogenicity in host [52]. Previously, *E. anguillarum* was identified as *E. tarda*. It was reclassified as *E. anguillarum*, a species within the genus *Edwardsiella* [51]. Since it was isolated from eels, this bacterium was named *E. anguillarum*. It has been reported in various fish species, including catfish (*Pangasianodon hypophthalmus*), tilapia (*Oreochromis* spp.), sharpsnout seabream (*Diplodus puntazzo*), and zebrafish (*Danio rerio*) [50]. *E. ictaluri*, a major pathogen in channel catfish (*Ictalurus punctatus*) and white catfish (*Ameiurus catus*), can cause diseases in various fish species [53]. Infected catfish show small red and white ulcers on their skin, petechial hemorrhage on the ventral side, and pimples between the eyes [43]. Previously, *E. tarda* was known as a main pathogen of freshwater and marine fish. It has been reported to infect more than 20 commercially important fish species in various regions, including the USA, Europe, and Asia [54]. However, it was found that isolates from fish previously identified as *E. tarda* were misclassified with the development of genetic investigation. Thus, the name *E. piscicida* was proposed [55]. Since its reclassification, *E. piscicida* has been isolated from various fish species in different countries [56]. More recently, *E. piscicida* infection was reported in marbled eels (*Anguilla marmorata*) cultured in Korea, causing mass mortality of over 65% [57].

The genus *Flavobacterium* within the family *Flavobacteriaceae* is known to cause bacterial diseases and has emerged as a significant concern in global aquaculture. The following three bacteria are recognized as serious pathogens in fish aquaculture, causing flavobacterial diseases: *F. columnare*, *F. psychrophilum*, and *F. branchiophilum* [58]. *F. columnare* causes columnaris disease in various freshwater fish [59]. Infected fish suffer from an acute to chronic infection of the gills, skin, and fins [59] and are unable to eat due to oral lesions such as mouth rot [59]. *F. psychrophilum* is the etiological agent of bacterial cold water disease in salmonids and rainbow trout (*Oncorhynchus mykiss*) fry syndrome in trout [60]. Its infection is characterized by saddle-like skin lesions near the dorsal fin, darkening of the fish, and necrosis of the mouth. *F. psychrophilum* was reported to cause 90% mortality in rainbow trout fry, presenting symptoms of anorexia, darkening of the caudal peduncle, and a distended abdomen [61]. Although it has been believed that *F. branchiophilum* is a causative agent of bacterial gill disease (BGD), environmental parameters and other bacteria also play crucial roles in BGD outbreaks [43].

The genus *Lactococcus* belongs to the family Streptococcaceae. Lactococcosis, a type of streptococcosis caused by *L. garvieae*, affects both freshwater and marine fish. In yellowtail (*Seriola quinqueradiata*), infected individuals exhibit damage to the kidneys, liver, intestine, and spleen, along with ascites accumulation. In rainbow trout, eye hemorrhaging is noted [43]. Infected marine fish display blood in the peritoneal cavity, a pale liver, and enteritis, though their kidneys remain unaffected [43,48].

*P. aeruginosa* is ubiquitous in marine environments, and its infections have been reported in both humans and fish [43]. *P. plecoglossicida* is the causative agent of bacterial hemorrhagic ascites disease in freshwater ayu fish (*Plecoglossus altivelis*). The principal clinical sign of *P. plecoglossicida* infection is bloody ascites. Lesions may also be observed in the kidney, liver, spleen, heart, intestine, and gills [43,48].

*S. iniae* is a zoonotic pathogen that infects both humans and fish [43,62]. In humans, streptococcosis is characterized by symptoms including cellulitis, endocarditis, meningitis, and arthritis [62]. As a fish pathogen, streptococcosis is recognized as a significant disease, particularly in tilapia, rainbow trout, coho salmon (*Oncorhynchus kisutch*), and yellowtail [62].

*V. anguillarum* is the causative agent of vibriosis, a lethal hemorrhagic septicemic disease affecting various aquatic organisms in both freshwater and saltwater environments, including economically significant species like Atlantic salmon (*Salmo salar*), rainbow trout, turbot (*Psetta maxima*), sea bass (*Dicentrarchus labrax*), and sea bream (*Sparus aurata*) [63,64]. Clinically, the disease manifests with symptoms such as weight loss, lethargy, red spots on the ventral skin, and swollen, dark skin lesions associated with ulcers and bleeding [63]. *V. harveyi*, known for its natural bioluminescence, affects a diverse range of fish and shellfish species [43]. Key symptoms include hemorrhaging, necrotizing enteritis, and gastroenteritis, leading to high mortality rates [43]. *V. harveyi* is also known as a causative agent of vibriosis in shrimp, leading to high mortality rates. Infected shrimp exhibit slow growth and decreased appetite [43]. *V. parahaemolyticus* is commonly found in marine and estuarine coastal environments and can cause foodborne gastroenteritis [65]. Outbreaks are often associated with the consumption of raw seafood, including crabs, fish, lobsters, oysters, shellfish, shrimp, and mussels [13]. *V. parahaemolyticus* can also cause significant mortality in shrimp aquaculture as the causative agent of acute hepatopancreatic necrosis disease (AHPND) [66]. *V. splendidus* is associated with skin ulceration syndrome in sea cucumbers (*Apostichopus japonicus*) [14].

### 2.2. Emergence of Antibiotic Resistance and Alternative Approaches

Antibiotics, among the most successful therapeutic interventions in medical history, are commonly used to reduce disease burden in aquaculture. They are applied prophylactically, therapeutically, metaphylactically, or indiscriminately to promote growth in aquaculture [67,68,69,70]. The availability and regulation of antibiotics in aquaculture vary significantly across countries and are controlled by regulatory systems in Europe, North America, and Japan [11,71,72]. However, their use is not adequately regulated in many developing countries, although these countries lead in global aquaculture production [70,72,73]. Additionally, the number of authorized antibiotics differs widely between countries: Vietnam authorizes the use of 30 antibiotics, whereas the UK and the USA authorize 5 and 4 antibiotics, respectively [70]. Moreover, fish farmers in several countries use antibiotics indiscriminately without identifying the diseases [74]. The indiscriminate and/or incorrect use of antibiotics increases the incidence of antibiotic-resistant bacteria, and this resistance can be transferred to other bacteria via horizontal gene transfer [75].

Global attention has focused on the presence of antibiotic residues in aquaculture products, which can be systemically toxic to consumers and devastatingly impact the normal flora [76]. Indeed, issues related to antibiotic residues have become some of the most frequent causes of detentions at international borders, leading to import refusals in the worst-case scenario [77,78].

The use of alternatives to conventional antibiotics for treating bacterial infections in aquaculture has been proposed, including vaccination, phages, probiotics, and prebiotics [11]. Among these alternatives, vaccination has been predominant [11]. Although vaccination technology appears promising, its actual use has been limited due to serious side effects such as impaired growth, inflammation, fibrous adhesions in internal organs, scarification, and pigment deposition [79,80,81,82]. Furthermore, vaccinating fish is labor intensive, time consuming, and often yields inconsistent results [11,18]. The efficacy of pro- and prebiotics remains uncertain, as their desirable effects are seldom reproducible [83]. Currently, the mechanism of action of pro- and prebiotics is poorly understood; thus, further research is needed on the interactions between hosts and microbes [11]. Additionally, phages have been successfully employed to manage bacterial infections in aquaculture and have garnered significant interest as an alternative to antibiotics. In this review, we present models of successful real-world applications of phages that aim to prevent and control major bacterial pathogens in aquaculture.

## 3. Phage Application in Aquatic Animals

Phage research has expanded its applications in aquaculture, particularly for economically significant species. Table 1 summarizes successful studies on controlling pathogenic bacteria in aquatic animals and seafood using phages, supported by evidence from in vivo animal experiments.

### 3.1. Biocontrol of Fish Diseases Using Phages

To date, phage research has been conducted continuously, mainly with bacterial species of greater interest in aquaculture, such as several *Aeromonas* and *Vibrio* species, as shown in Table 1. Especially the genus *Aeromonas*, the first reported target bacteria for phage application in aquaculture, has been continuously studied for more than 50 years. Phage therapy against fish bacterial disease was reported for the first time in 1981, which demonstrated the efficacy of the phage AH1 in a loach model against *A. hydrophila* [17]. It was reported that AH1 eliminated the pathogenicity of bacteria when pathogenic bacteria were infected with AH1 before injection in fish [17]. In a previous report, φ2 and φ5 intraperitoneal (IP) injection improved the survival rates of catfish from 18.3% to 100% against *A. hydrophila* [15]. Akh-2 application by immersion reduced the cumulative mortality rate of loach from 100% to 56.67% against *A. hydrophila* [16]. Successful phage therapy by IP injection and oral administration (feeding) was reported in loach against *A. hydrophila* resistant to multiple antibiotics. For example, pAh1-C reduced cumulative mortality rates from 100% to 43.33% by IP injection and from 95.83% to 46.67% by oral administration. In addition, pAh6-C reduced cumulative mortality rates from 100% to 16.67% by IP injection and from 95.83% to 26.67% by oral administration [18]. Phages have demonstrated efficacy against *A. salmonicida* infection. For example, PAS-1 improved survival rate from 0% to 26.7% by intramuscular injection in rainbow trout [19]. AS-A reduced cumulative mortality rate from 36% to 0% by immersion in Senegalese sole [20]. HER 110 reduced cumulative mortality rate from 100% to 10% by immersion in brook trout [21].

The prophylactic use of phages against edwardsiellosis has been documented. ETP-1 enhanced survival rates from 18% to 68% by immersion prior to *E. tarda* challenge in zebrafish [22]. Phage biocontrol of *Flavobacterium* has also been reported [23,24].

When applied against columnaris disease by immersion in rainbow trout and zebrafish, FCL-2 increased survival rates from 8.3% to 50% in rainbow trout and from 0% to 60% in zebrafish [23]. Additionally, two types of siphovirus, 1H and 6H, decreased cumulative mortality rates against *F. psychrophilum* infection by IP injection from 80% to 67% and 47% in rainbow trout and from 13% to 0% and 6% in salmon, respectively [24].

Phage biocontrol via two different administration methods was demonstrated against lactococcosis in yellowtail [25]. PLgY-16 raised the survival rate from 45% to 90% via IP injection and decreased cumulative mortality from 65% to 10% through oral administration [25]. When two distinct phages, PPpW-3 and PPpW-4, were orally administered alone and in combination against *P. plecoglossicida* in ayu, they reduced cumulative mortality rates from 93.3% to 53.3%, 40.0%, and 20.0%, respectively [26].

Studies on phage biocontrol against streptococcosis were conducted via IP injection. HN48 significantly improved the survival rate from 0% to 60% against *S. agalactiae* in Nile tilapia [27]. *S. iniae* phages, PSiJ 31, 32, 41, and 42 demonstrated enhanced efficacy, with survival rates reaching up to 90%, indicating increased effectiveness of the phage cocktail [28].

Phage biocontrol of *Vibrio anguillarum* has been reported. CHOED significantly enhanced the survival rate of Atlantic salmon from less than 10% to 100% via immersion [29]. VP-2 reduced the cumulative mortality in zebrafish larvae from 17% to 2% through immersion [30].

### 3.2. Phage Biocontrol of Shrimp Diseases

Since shrimp lack a vertebrate-like adaptive immune system, there are no viable remedies for shrimp bacterial diseases except for antibiotics. This underlines the potential of phage therapy in shrimp aquaculture, where reliance on antibiotics is prohibited. Phage application for controlling shrimp disease has focused on vibriosis, as depicted in Table 1. Given the characteristics of shrimp aquaculture, phages have been applied by immersion to combat shrimp diseases. It has been reported that vibrio phages A, VHM1, VHM2, VHP6b, Viha10, and Viha8 enhanced the survival rates of shrimp larvae, including black tiger shrimp larvae, when administered via immersion [31,32,34,35]. Additionally, there is evidence of phage application in juvenile marine shrimp against AHPND, with pVp-1 significantly reducing cumulative mortality through either immersion or oral administration (feeding) [37]. Notably, the administration of pVp-1 through feeding reduced the cumulative mortality rate from 100% to 0% in juvenile marine shrimp [37].

### 3.3. Phage Biocontrol of Shellfish Diseases

In shellfish aquaculture, phage biocontrol has been investigated against *Vibrio* spp., including *V. harveyi* and *V. parahaemolyticus* (Table 1). The phage vB_VhaS-tm was reported to enhance the survival rate of abalone larvae through immersion [33]. Additionally, VP10 effectively reduced the growth of *V. parahaemolyticus* to undetectable levels in blue mussels by immersion [13]. Moreover, surface inoculation was identified as a unique method of phage application, successfully reducing the growth of *V. parahaemolyticus* from 10^6^ CFU/g to 10 CFU/g on oyster surfaces [36].

### 3.4. Phage Biocontrol of Other Marine Invertebrates

Two *myoviruses*, PVS-1 and PVS-2, along with a *siphovirus*, PVS-3, were investigated for their effectiveness against *V. splendidus* infection in sea cucumbers, as indicated in Table 1 [14]. These studies demonstrated that phage application significantly improved survival rates, rising from 18% to 82% through oral feeding and from 20% to 80% through coelomic injection [14].

## 4. Conclusions and Perspectives

Phage research for both the treatment and prophylaxis of bacterial infections has been conducted in the fields of human and veterinary medicine, agriculture, and the food sectors, extending into aquaculture [84]. The rising incidence of antibiotic resistance poses a threat to aquaculture, indicating that the imprudent use of antibiotics is exacerbating this issue [18,85]. As the number of authorized antibiotics in aquaculture is limited, difficult situations arise with no available remedies for antibiotic-resistant strains. Coupled with the antibiotic resistance issue, the presence of antibiotic residues has raised serious concerns in the aquaculture industry, as it causes friction during the international trade of aquaculture products, particularly at the borders of the largest fish markets in the European Union, the United States, and Japan, resulting in significant economic losses for exporting countries. Consequently, various alternatives have been proposed for use in aquaculture, including vaccination, phages, probiotics, and prebiotics. However, the administration of vaccines is limited in aquaculture due to various side effects and cost considerations. Pro- and prebiotics have not been consistently effective. As an alternative approach, phages have been extensively researched in aquaculture for several decades, highlighting their potential as biocontrol agents for sustainable aquaculture. Although phages have been considered an alternative to traditional antibiotics for treating diseases in aquatic animals, key points should be considered for their practical application in aquaculture: (i) The efficacy of phages must be supported by in vivo experiments. Evidence has shown that in vitro results may not be indicative of outcomes in in vivo trials due to unpredictable factors [86]. To date, numerous publications have documented the protective efficacy of phages against diseases in aquatic animals, underscoring their promising potential. Nevertheless, research results based on in vivo studies, even under controlled laboratory conditions, remain insufficient. To fulfill the needs of aquaculturists, in vivo evidence is essential, and quantitative efforts must be made to obtain in vivo data [84]. Despite the necessity of animal experiments, a considerable number of phage researchers face significant challenges in conducting in vivo experiments at their facilities. As such, collaborative research is highly recommended, as there are several research groups with resources and facilities approved by the Institutional Committee for the Care and Use of Animals (IACUC). (ii) In any event, ensuring that phages are safe for human and aquatic animals is non-negotiable. Although there is no scientific evidence to suggest that phages pose a safety problem, the issue of safety remains a significant barrier within legal and regulatory frameworks [10]. An intensive investigation of the phage genome sequence will mitigate potential risks raised by regulatory authorities. Additionally, it is undeniable that further extensive research is necessary for the regulatory approval of phages based on in vivo studies. (iii) Phage administration should be economical and feasible. While injections have generally shown successful protective effects in phage therapy, this method may not be practical on a larger scale, such as on an aquaculture farm [18,84]. Establishing a strategy for phage application that considers the target animal species and population size at the aquaculture farm would be beneficial.

There has been significant public interest in microbial contamination as a major cause of foodborne outbreaks [10]. Specifically, the risk of cross-contamination during seafood processing is considerable. The risk of both waterborne and foodborne outbreaks in seafood, taking into account consumption patterns, should be rigorously investigated. Thus, increased phage research is needed to control waterborne bacterial infections. Previous studies have shown that *Shigella* phages can effectively control the growth of well-known waterborne pathogens such as *S. flexneri*, *S. boydii*, and *S. sonnei* [87,88,89,90]. Moreover, it is advisable to explore diverse application strategies for phages. Phages are versatile and ideal for both prophylactic and therapeutic purposes. Phage therapy offers the advantage of being applicable at various infection stages, although the timing of phage treatment often significantly affects the efficacy of protection. For bacterial infections with very rapid progression, careful attention to the timing of phage application is crucial, as delays in phage treatment have been shown to decrease protective efficacy [37]. For sustainable development in aquaculture, the environmental impact is a primary concern. Continuous interest in recirculating aquaculture systems (RASs) has been maintained, not as a new technique but as an established one, because RASs can decrease water intake by reusing water. However, controlling diseases in RASs remains challenging; pathogenic bacteria can spread throughout the closed system, potentially leading to uncontrollable outbreaks. Phage therapy could be especially beneficial in RASs, as phages can survive long periods within the system, and recirculation allows them to interact freely with target bacteria. It has been documented that components of RASs (e.g., the biofilter) positively affect phage persistence, with phages surviving in RASs for up to three weeks without additional treatment [91]. Given that the use of antibiotics impacts microbiome composition by disrupting microbial balance in RASs, phage therapy emerges as an ideal method to control bacterial infections, as it specifically targets pathogens while preserving beneficial bacteria. It is applicable to various animal species. For instance, pVp-1 has demonstrated both prophylactic and therapeutic effects on vibriosis in oyster, shrimp, and mouse experimental models [36,37,92]. However, phage resistance has been observed, raising concerns that may limit phage therapy’s positive impact [84,93]. Therefore, a phage cocktail is recommended to enhance the efficacy of phage therapy.

## Figures and Tables

**Table 1:** In vivo phage trials conducted for the biocontrol of pathogens in aquatic animals and seafood.

Pathogen	Disease	Animal or Seafood Species	Name of Phage (Morphology) ^1^	Method of Application ^2^	Outcome	Reference
*Aeromonas hydrophila*	Septicemia	Catfish (*Pangasianodon hypophthalmus*)	φ2 and φ5 (*Myoviridae*)	I.P. injection	Improved survival rates from 18.3% to 100%	[15]
		Loach (*Misgurnus anguillicaudatus*)	Akh-2 (*Siphoviridae*)	Immersion	Reduced cumulative mortality rates from 100% to 56.67%	[16]
		Loach (*Misgurnus anguillicaudatu*)	AH1 (n.d.)	Infection with phage before injection in fish	Pathogenicity eliminated following phage infection	[17]
		Loach (*Misgurnus anguillicaudatu*)	pAh1-C, pAh6-C (*Myoviridae*)	I.P. injection and oral administration via feeding	Reduced cumulative mortality rates I.P. injection: from 100% to 43.33% using pAh1-C and to 16.67% using pAh6-C Oral administration: from 95.83% to 46.67% with pAh1-C and to 26.67% with pAh6-C	[18]
*A. salmonicida*	Furunculosis	Rainbow trout (*Oncorhynchus mykiss*)	PAS-1 (*Myoviridae*)	I.M. injection	Increase in survival rates from 0% to 26.7%	[19]
		Senegalese sole (*Solea senegalensis*)	AS-A (*Myoviridae*)	Immersion	Decrease in cumulative mortality rates from 36% to 0%	[20]
		Brook trout (*Salvelinus fontinalis*)	HER 110 (*Myoviridae*)	Immersion	Decrease in total mortality rates from 100% to 10%	[21]
*Edwardsiella tarda*	Edwardsiellosis	Zebrafish (*Danio rerio*)	ETP-1 (*Podoviridae*)	Immersion prior to bacterial challenge	Survival rates improved from 18% to 68%	[22]
*Flavobacterium columnare*	Columnaris disease	Rainbow trout and zebrafish	FCL-2 (*Myoviridae*)	Immersion	Improved survival rates Rainbow trout: increased from 8.3% to 50% Zebrafish: increased from 0% to 60%	[23]
*F. psychrophilum*	Rainbow trout fry syndrome and cold water disease	Rainbow trout and Atlantic salmon (*Salmo salar*)	1H, 6H (*Siphoviridae*)	I.P. injection	Reduced cumulative mortality rates Trout: decreased from 80% to 67% (1H), 47% (6H) Salmon: from 13% to 0% (1H), 6% (6H)	[24]
*Lactococcus garvieae*	Lactococcosis	Yellowtail (*Seriola quinqueradiata*)	PLgY-16 (*Siphoviridae*)	I.P. injection and oral administration (feeding)	I.P. injection: improved survival rates from 45% to 90% Oral administration: reduced cumulative mortality from 65% to 10%	[25]
*Pseudomonas plecoglossicida*	Bacterial hemorrhagic ascites disease	Ayu (*Plecoglossus altivelis*)	PPpW-3 (*Myoviridae*), PPpW-4 (*Podoviridae*)	Oral administration (feeding)	Reduced cumulative mortality rates from 93.3% to 53.3% for PPpW-3, 40.0% for PPpW-4, and 20.0% for PPpW-3/W-4	[26]
*Streptococcus agalactiae*	Streptococcosis	Nile tilapia (*Oreochromis niloticus*)	HN48 (n.d.)	I.P. injection	Improved survival rates from 0% to 60%	[27]
*S. iniae*	Streptococcosis	Japanese flounder (*Paralichthys olivaceus*)	PSiJ31, 32, 41, 42 (*Siphoviridae*)	I.P. injection	Improved survival rates from 0% to 28 or 33% (combined usage of PSiJ31 and 32); from 0% to 48, 70, or 90% (combined usage of PSiJ31, 32, 41, and 42)	[28]
*Vibrio anguillarum*	Hemorrhagic septicemia	Atlantic salmon	CHOED (n.d.)	Immersion	Survival rates improved from less than 10% to 100%	[29]
	Vibriosis	Zebrafish larvae	VP-2 (n.d.)	Immersion	Cumulative larval mortality rates reduced from 17% to 2%	[30]
*V. harveyi*	Luminescent vibriosis	Shrimp (*Penaeus monodon*) larvae	A (*Siphoviridae*)	Immersion	Larval survival rates improved from 17% to 86%	[31]
		Shrimp larvae	VHM1, VHM2 (*Myoviridae*) VHS1 (*Siphoviridae*)	Immersion	Larval survival rates improved from 26.6% to 86.6%	[32]
		Abalone (*Haliotis laevigata*)	vB_VhaS-tm (*Siphoviridae*)	Immersion	Larval survival rates improved from 0% to 70%	[33]
		Black tiger shrimp (*Litopenaeus monodon*) larvae	VHP6b (*Siphoviridae*)	Immersion	Cumulative larval mortality rates reduced from 70% to 20%	[34]
		Shrimp larvae	Viha10, Viha8 (*Siphoviridae*)	Immersion	Larval survival rates improved from 65% to 88%	[35]
*V. parahaemolyticus*		Blue mussel (*Mytilus edulus*)	VP10 (n.d.)	Immersion	Reduction in bacterial growth to undetectable levels	[13]
		Oyster (*Crassostrea gigas*)	pVp-1 (*Siphoviridae*)	Immersion, surface inoculation	Reduction in bacterial growth from 10^6^ CFU/g to 10 CFU/g	[36]
	Acute hepatopancreatic necrosis disease	Marine shrimp (*P. vannamei*)	pVp-1 (*Siphoviridae*)	Immersion, oral administration	Reduced cumulative mortality rates from 100% to 0%	[37]
*V. splendidus*	Vibriosis	Sea cucumber (*Apostichopus japonicus*)	PVS-1, PVS-2 (*Myoviridae*) PVS-3 (*Siphoviridae*)	Oral administration (feeding) and coelomic injection	Survival rates increased from 18% to 82% through feeding and from 20% to 80% via coelomic injection	[14]

^1^ n.d. (not determined); ^2^ I.P. (intraperitoneal); I.M. (intramuscular).
